# Inflammatory geriatric nutritional risk index stratified the survival of older adults with cancer sarcopenia

**DOI:** 10.1002/cam4.5427

**Published:** 2022-11-29

**Authors:** Guo‐Tian Ruan, Hai‐Lun Xie, He‐Yang Zhang, Qi Zhang, Xi Zhang, Yi‐Zhong Ge, Chun‐Lei Hu, Meng Tang, Meng‐Meng Song, Xiao‐Wei Zhang, Ming Yang, Kai‐Ying Yu, Yi‐Zhen Gong, Li Deng, Han‐Ping Shi

**Affiliations:** ^1^ Department of Gastrointestinal Surgery/Department of Clinical Nutrition Beijing Shijitan Hospital, Capital Medical University Beijing China; ^2^ Key Laboratory of Cancer FSMP for State Market Regulation Beijing China; ^3^ Department of Gastrointestinal Surgery Guangxi Medical University Cancer Hospital Nanning People's Republic of China

**Keywords:** GNRI, reduced intake, reduced physical activity, systemic inflammation

## Abstract

**Background:**

Aging is accompanied by muscle loss. In older adults with cancer sarcopenia (OACS), systemic inflammation, reduced food intake, and reduced physical activity led to a poor prognosis. This study was to investigate the prognostic ability of the inflammatory Geriatric Nutritional Risk Index (GNRI), which combines patient's inflammation, diet status, and physical activity status to predict overall survival of OACS.

**Methods:**

This prospective multi‐center study enrolled 637 OACS, with an average age of 72.78 ± 5.98 years, of which 408 (64.1%) were males. We constructed the Inflammatory Functional Prognostic Index (IFPI) of OACS based on inflammatory GNRI scores, reduced food intake, and reduced physical activity. According to the IFPI, OACS was divided into high‐, moderate‐, and low‐risk groups. Univariate and multivariate survival analyses analyzed the prognostic ability of the clinical parameters.

**Results:**

Compared with OACS with a high GNRI score, the 1‐, 3‐, and 5‐year hazard ratios (95% confidence interval) of OACS with a low GNRI score was 1.816 (1.076–3.063), 1.678 (1.118–2.518), and 1.627 (1.101–2.407), respectively. This result was consistent with that of the calibration curve. The subgroup analysis showed that the low GNRI score had a significant positive relation with patients with gastrointestinal cancer (*P*_interaction_ < 0.001). Notably, the survival analysis of IFPI showed that the mortality risk of moderate‐ and high‐risk patients was 1.722‐and 2.509‐fold higher, respectively, than that of low‐risk patients.

**Conclusion:**

The GNRI score was a short‐term and long‐term inflammatory prognostic indicator for OACS. The IFPI score could improve patient survival prediction.

## 1 INTRODUCTION

Sarcopenia (or muscle wasting) has been considered as an easily available option to assess the overall health and weakness of patients. The European Working Group on Sarcopenia in Older People^1^ and the Asian Working Group for Sarcopenia (AWGS)^2^ suggested that the two dimensions of muscle strength loss and functional impairment should be added to the definition of skeletal sarcopenia. Skeletal sarcopenia is associated with a series of poor outcomes, including increased postoperative complications,^3^ long hospital stays,^3^ increased 30‐day postoperative and hospital mortality,^4^ and increased dose‐limiting toxicity.^5^ Sarcopenia is an outcome which is caused by old age; it may also be related to chronic diseases, including cancer.^6^ Sarcopenia is also an objective indicator of cancer cachexia, which is defined as a condition caused by the systemic inflammation of cancer and is associated with a poor outcome.^7^ The lack of activity, age‐related factors, anorexia, inflammation, and nutritional imbalance affect skeletal muscle changes.^8^ Indeed, the comprehensive effects of aging, cancer, sarcopenia, diet status, and physical activity status on the prognosis of patients' needs further attention. Aging is a global problem, and as the proportion of elderly individuals continues to rise, it is necessary to formulate treatment policies for different age groups. Older adults with cancer sarcopenia (OACS) usually have reduced food intake and insufficient physical activity. This study mainly shows the inflammation and functional prognosis analysis of these patients.

Currently, the nutritional risk index applied to elderly individuals, the Geriatric Nutritional Risk Index (GNRI), is mainly including the albumin level and the ratio of current/usual body weight.^9^ It is difficult to get the weight data history of older adults. Therefore, replacing history weight with ideal weight in the GNRI formula can improve the assessment ability of the nutritional status of older adults. Additionally, as a comprehensive indicator, the GNRI is not only closely related to the nutritional status but also significantly related to skeletal muscle quality and immunity.^10^ Hypoalbuminemia is a reflection or hallmark of systemic inflammation. Inflammatory cytokines, such as interleukin (IL)‐1 and ‐6, can reduce liver albumin synthesis.^11^, ^12^ Systemic inflammation is the main cause of malnutrition and cachexia in cancer patients.^13^ In addition, systemic inflammation is closely associated to tumor progression and metastasis.^14^ Patients with cancer are prone to anorexia, leading to reduced food intake. Most patients with cancer have symptoms that lead to inadequate nutritional intake and excessive protein consumption, which are often caused by anorexia, early satiety, and dysphagia. The imbalance of energy leads to a decrease in muscle mass. Furthermore, the decline in physical activity of patients with cancer also affects their muscle strength. The lack of exercise can cause muscle protein degradation, which can easily induce sarcopenia and fatigue. Thus, our study aimed to investigate the combined prognostic effects of systemic inflammation, reduced food intake, and reduced physical activity on overall survival (OS) of OACS.

## 2 MATERIALS AND METHODS

### 2.1 Patient selection and study design

This was a prospective, observational cohort study conducted at multiple medical hospital centers in China (Table S1). The data were collected from July 2013 to December 2019. The study protocol (registration number: ChiCTR1800020329) was approved by the independent ethics committees/institutional review committees of all participating centers. The study was conducted in accordance with the Declaration of Helsinki, and all participants signed informed consent forms. Adult patients (≥18 years) who were diagnosed with cancer and were hospitalized for at least 2 days met the inclusion criteria of the study. The exclusion criterion was that patients were unable to self‐response the questionnaire.

In the primary cohort, there were 9727 patients diagnosed with cancer, and 1204 patients with cancer sarcopenia were identified by screening using the diagnostic criteria for sarcopenia. Finally, 637 patients with cancer sarcopenia aged 65 years or older were enrolled in our final study. The screened details were shown in the flowchart (Figure S1). The mean age (standard deviation; SD) of the enrolled patients was 72.78 (5.98) years, and 408 (64.1%) were males.

### 2.2 Data collection and study outcomes

Trained interviewers conducted a questionnaire survey of all participants through face‐to‐face interviews within 48 hours of enrollment and collected the main study data. Anthropometric measurements were also performed by well‐trained technicians. All interviewers and technicians had received the survey manual, multimedia materials, and patient simulation training and were required to pass the training test before the formal survey. In general, patients with a total of nine major solid cancers were included in our study, including lung, esophageal, gastric, colorectal, pancreatic, hepatobiliary, breast, utero‐ovarian, nasopharyngeal cancer, and other cancer subtypes. Collected data included socio‐demographics (age, sex, height, weight, and body mass index [BMI]), comorbidities, daily habits (smoking, alcohol consumption, and tea consumption), the tumor‐node‐metastasis (TNM) stage, radical resection (yes vs. no), neoadjuvant chemoradiotherapy history, postoperative chemoradiotherapy history, the European Organization for Research and Treatment of Cancer Quality of Life Questionnaire‐Core 30 (EORTC QLQ‐C30) results, Karnofsky Performance Status (KPS), 30‐day mortality, diet status, physical activity status, and nutritional intervention history. Clinical laboratory indicators, including serum total protein, serum albumin, alanine transaminase [ALT], aspartate aminotransferase [AST], and hemoglobin levels and white blood cell [WBC], lymphocyte, and neutrophil counts were also collected. The assessment of diet status and physical activity status in OACS was in the form of a questionnaire, and the assessment of diet status and physical activity status was determined based on the patient's current perception of subjective changes in diet status and physical activity status (declined or no change). It is worth noting that the height and weight were measured by repeated measurements with the patient wearing a light gown and socks, and that laboratory test samples were taken within 48 hours prior to admission without any treatment intervention.

The BMI was calculated as weight (kg)/height^2^ (m^2^). The prognostic nutritional index (PNI) score was calculated as albumin (g/L) + 0.005 × lymphocyte count (per mm^3^). The GNRI was calculated as 1.489 × serum albumin (g/L) + 41.7 × (present body weight [PBW, kg]/ideal body weight [IBW, kg]). The IBW (kg) was calculated using the Lorentz equation: for men = height (cm) − 100 − ([height − 150]/4); for women = height (cm) − 100 − ([height − 150]/2.5).^9^ When the PBW exceeded the IBW, weight/ideal weight = 1.^9^ The four prognostic risk levels defined by Boellanie et al, according to the GNRI score, were as follows: severe risk (GNRI score < 82), moderate risk (GNRI score 82–92), low risk (GNRI score 92–98), and no risk (GNRI score > 98).^9^ Additionally, we also calculated the best cut‐point value related to survival from the largest selected rank statistic in the “maxstat” R package, where the PNI score was 41.45 and GNRI score was 97.77 (Figure S2).

The patient follow‐up data was obtained from the date of initial hospitalization to the end of the follow‐up. Follow‐up was conducted by reviewing outpatient or inpatient follow‐up records, as well as regular telephone follow‐ups. OS was calculated as the time from initial diagnosis to death from any cause or the time of the last follow‐up.

### 2.3 Assessment of cancer sarcopenia

According to the 2019 AWGS diagnostic consensus on sarcopenia, a diagnosis was made per low muscle strength (hand grip strength, HGS) and a low appendicular skeletal muscle index (ASMI) score.^15^ The measurement of HGS was performed as follows: The handle was adjusted according to the size of the patient's hand. The patient sat upright with his arms resting on the armrests of the chair and his elbow bent at 90°. The patient was instructed to hold the handle with maximum force within 3 seconds. The test was performed three times in a row, and the maximum HGS was recorded.

The SMI was assessed through an validated equation of the Chinese population: SMI = 0.193 × bodyweight (kg) + 0.107 × height(cm) − 4.157 × sex − 0.037 × age (year) − 2.631.^16^ For sex, male was represented by a value of 1, while female was represented by a value of 2.^16^, ^17^, ^18^ The SMI equation model result was in good agreement with the results of double X‐ray absorptiometry (Adjusted R^2^ = 0.90, standard error of estimate = 1.63 kg).^16^ After evaluating SMI value, the ASMI score was a combination of the SMI value and height, namely SMI/height^2^(m^2^).^17^, ^18^ According to the previous description, the cut‐off value of low muscle mass was defined according to the lowest 20% ASMI score in the study population.^17^, ^18^ The low ASMI score classification criteria were 7.0 kg/m^2^ for men and 5.4 kg/m^2^ for women. The grading standard for a low HGS was 28 kg for men and 18 kg for women.^15^

### 2.4 Statistical analysis

Descriptive statistics were used to summarize patient demographic characteristics, disease characteristics, laboratory indicators, and treatment methods. Continuous variable data were represented by mean ± SD or median ± interquartile range (IQR), and categorical variables were showed in absolute numbers or proportions (%). Differences between groups were assessed by the analysis of categorical data using Pearson's chi‐square test, continuous data with normal distribution by using the one‐way analysis of variance, and continuous data with abnormal distribution by using the Mann–Whitney *U* test. The Cox proportional model was used to estimate the crude and adjusted hazard ratio (HR) and 95% confidence interval (CI) of mortality. The selection of prognostic adjustment factors was based on clinical parameters with prognostic significance in the univariate survival analysis (Table S2). The adjusted model included sex, radical resection, the TNM stage, the BMI, the KPS, postoperative chemoradiotherapy history, the neutrophil count, the WBC count, AST levels, ALT levels, serum albumin levels, comorbid disease history, family history of cancer, hemoglobin levels, 30‐day mortality, diet status, physical activity status, and the PNI score. We constructed the inflammatory functional prognostic index (IFPI), which was composed of the inflammation‐related GNRI and function‐related reduced intake and reduced physical activity. We defined the four risk groups of the GNRI as follows: no risk, 0‐point; low risk, 1‐point; moderate risk, 2‐points; and severe risk, 3‐points; reduced food intake: yes, 1‐point; no, 0‐point; and reduced physical activity: yes, 1‐point; no, 0‐point. All statistical analyses in this study were performed using the R platform. A two‐tailed *p* less than 0.05 represented statistical significance.

## 3 RESULTS

### 3.1 Baseline characteristics

The median survival time of this study was 20.5 months (95% CI, 18.1–26.1). We observed that the overall mortality (rate) of patients in 5 years was 359 (64.5%), which equated to 312 mortality events per 1000 patient‐years. In the GNRI subgroup, the number of patients in the no risk, low risk, moderate risk, and severe risk groups was 88 (13.8%), 142 (22.3%), 244 (38.3%), and 163 (25.6%), respectively. Other than the subgroups based on age, comorbidities, family history of cancer, alcohol consumption, tea consumption, radical resection, neoadjuvant chemoradiation, and 30‐day mortality, all variables showed differences among the GNRI subgroups (*p* < 0.05). Meanwhile, among our patients in the KPS or EORTC QLQ‐C30 subgroups, there was a downward trend as the GNRI score decreased. The number of patients in the reduced intake and reduced physical function subgroups also increased with the decline in the GNRI score (Table 1).

**TABLE 1:** Demographic and clinical characteristics

Characteristics	Overall	Stratified by scored‐GNRI				
	Patients (*n*, %)	No risk (98~)	Low risk (92 ~ 98)	Moderate risk (82 ~ 98)	Severe risk (~82)	*p* value
	(*n* = 637)	(*n* = 88)	(*n* = 142)	(*n* = 244)	(*n* = 163)	
Age, years, (mean [SD])	72.78 (5.98)	72.43 (5.84)	72.43 (5.95)	72.92 (5.87)	73.08 (6.25)	0.724
Age, *n* (%)						
65–70 years	264 (41.4)	36 (40.9)	64 (45.1)	98 (40.2)	66 (40.5)	0.800
>70 years	373 (58.6)	52 (59.1)	78 (54.9)	146 (59.8)	97 (59.5)	
Sex, *n* (%)						
Male	408 (64.1)	38 (43.2)	83 (58.5)	169 (69.3)	118 (72.4)	<0.001
Female	229 (35.9)	50 (56.8)	59 (41.5)	75 (30.7)	45 (27.6)	
Sites of cancer, *n* (%)						
Lung cancer, *n* (%)	148 (23.2)	24 (27.3)	30 (21.1)	58 (23.8)	36 (22.1)	0.011
Gastric cancer, *n* (%)	128 (20.1)	10 (11.4)	26 (18.3)	48 (19.7)	44 (27.0)	
Colorectal cancer, *n* (%)	158 (24.8)	29 (33.0)	33 (23.2)	56 (23.0)	40 (24.5)	
Esophageal cancer, *n* (%)	74 (11.6)	6 (6.8)	18 (12.7)	35 (14.3)	15 (9.2)	
Hepatobiliary cancer, *n* (%)	18 (2.8)	2 (2.3)	4 (2.8)	4 (1.6)	8 (4.9)	
Pancreatic cancer, *n* (%)	20 (3.1)	2 (2.3)	4 (2.8)	9 (3.7)	5 (3.1)	
Breast cancer, *n* (%)	21 (3.3)	9 (10.2)	6 (4.2)	4 (1.6)	2 (1.2)	
Utero ovarian cancer, *n* (%)	16 (2.5)	3 (3.4)	6 (4.2)	6 (2.5)	1 (0.6)	
Nasopharyngeal cancer, *n* (%)	18 (2.8)	0 (0.0)	5 (3.5)	8 (3.3)	5 (3.1)	
Other cancer subtypes, *n* (%)	36 (5.7)	3 (3.4)	10 (7.0)	16 (6.6)	4 (2.5)	
Comorbid disease(s), yes, *n* (%)						
0	361 (56.7)	45 (51.1)	77 (54.2)	150 (61.5)	89 (54.6)	0.468
1	174 (27.3)	26 (29.5)	42 (29.6)	60 (24.6)	46 (28.2)	
2	66 (10.4)	13 (14.8)	11 (7.7)	23 (9.4)	19 (11.7)	
3 or more	36 (5.7)	4 (4.5)	12 (8.5)	11 (4.5)	9 (5.5)	
Family history of cancer, yes, *n* (%)	82 (12.9)	10 (11.4)	18 (12.7)	34 (13.9)	20 (12.3)	0.923
Smoking, yes, *n* (%)	317 (49.8)	33 (37.5)	64 (45.1)	126 (51.6)	94 (57.7)	0.012
Alcohol consumption, yes, *n* (%)	114 (17.9)	9 (10.2)	24 (16.9)	53 (21.7)	28 (17.2)	0.107
Tea consumption, *n* (%)	158 (24.8)	17 (19.3)	29 (20.4)	69 (28.3)	43 (26.4)	0.197
BMI, kg/m^2 (mean [SD])	18.69 (1.91)	20.10 (1.52)	19.53 (1.38)	18.48 (1.71)	17.53 (1.98)	<0.001
BMI, kg/m^2, *n* (%)						
≥18.5	374 (58.7)	78 (88.6)	114 (80.3)	132 (54.1)	50 (30.7)	<0.001
<18.5	263 (41.3)	10 (11.4)	28 (19.7)	112 (45.9)	113 (69.3)	
TNM stage, *n* (%)						
I	45 (7.1)	15 (17.0)	7 (4.9)	16 (6.6)	7 (4.3)	0.002
II	127 (19.9)	10 (11.4)	30 (21.1)	48 (19.7)	39 (23.9)	
III	162 (25.4)	29 (33.0)	36 (25.4)	64 (26.2)	33 (20.2)	
IV	303 (47.6)	34 (38.6)	69 (48.6)	116 (47.5)	84 (51.5)	
Radical resection, yes, *n* (%)	175 (27.5)	29 (33.0)	37 (26.1)	58 (23.8)	51 (31.3)	0.227
Neoadjuvant chemoradiotherapy, yes, *n* (%)	17 (2.7)	2 (2.3)	3 (2.1)	7 (2.9)	5 (3.1)	0.948
Postoperative chemoradiotherapy, yes, *n* (%)	278 (43.6)	44 (50.0)	62 (43.7)	107 (43.9)	65 (39.9)	0.495
EORTC QLQ‐C30	49.05 (9.60)	53.00 (8.71)	50.57 (9.20)	49.14 (9.21)	45.44 (9.80)	<0.001
KPS (mean [SD])	49.65 (18.19)	84.32 (14.99)	82.46 (14.98)	78.85 (18.09)	72.58 (20.63)	<0.001
Serum total protein (g/L) (mean [SD])	64.92 (7.87)	71.77 (7.41)	68.65 (4.96)	64.58 (6.11)	58.49 (7.52)	<0.001
Serum albumin (g/L) (mean [SD])	35.71 (5.72)	43.58 (3.22)	39.44 (1.98)	35.27 (2.52)	28.87 (4.11)	<0.001
Hemoglobin (g/L) (mean [SD])	112.36 (21.80)	125.08 (14.40)	119.91 (22.48)	112.83 (18.24)	98.21 (21.82)	<0.001
WBC (×10^9/L) (mean [SD])	6.95 (3.41)	6.23 (3.52)	6.45 (3.10)	6.68 (2.78)	8.19 (4.10)	<0.001
Neutrophils (×10^9/L) (mean [SD])	4.86 (3.28)	3.89 (3.20)	4.22 (2.89)	4.66 (2.67)	6.25 (4.00)	<0.001
Lymphocytes (×10^9/L) (mean [SD])	112.36 (21.80)	125.08 (14.40)	119.91 (22.48)	112.83 (18.24)	98.21 (21.82)	<0.001
AST, <40 U/L, *n* (%)	58 (9.1)	2 (2.3)	9 (6.3)	24 (9.8)	23 (14.1)	0.010
ALT, <50 U/L, *n* (%)	90 (14.1)	6 (6.8)	12 (8.5)	35 (14.3)	37 (22.7)	<0.00
30‐day mortality, yes, *n* (%)	20 (3.1)	1 (1.1)	2 (1.4)	7 (2.9)	10 (6.1)	0.059
Reduced intake, *n* (%)	381 (59.8)	35 (39.8)	78 (54.9)	147 (60.2)	121 (74.2)	<0.001
Reduced physical function, *n* (%)	336 (52.7)	33 (37.5)	63 (44.4)	130 (53.3)	110 (67.5)	<0.001
Nutritional intervention, yes, *n* (%)	180 (28.3)	12 (13.6)	37 (26.1)	59 (24.2)	72 (44.2)	<0.001
PNI (mean [SD]), (Kg)	42.74 (7.57)	51.29 (5.61)	46.85 (4.23)	42.05 (4.38)	35.58 (7.54)	<0.001

Abbreviations: ALT, alanine transaminase; AST, aspartate aminotransferase; BMI, body mass index; EORTC QLQ‐C30, The European O‐rganization for research and treatment of cancer quality of life questionnare‐core 30; GNRI, Geriatric Nutritional Risk Index; KPS, Karnofsky performance status; PNI, prognostic nutritional index; WBC, white blood cells.

### 3.2 Analysis of the prognostic ability and distribution of the GNRI score

**FIGURE 1:**
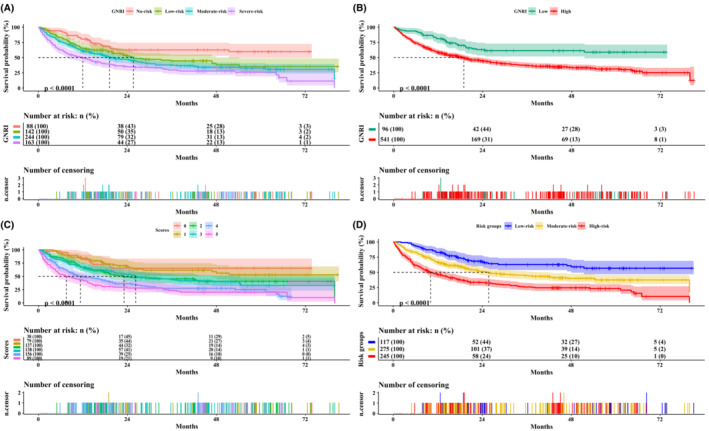
The Kaplan–Meier survival curves of OS in OACS. (A) GNRI risk groups; (B) the GNRI score; (C) the IFPI score; (D) IFPI risk groups. GNRI, geriatric nutritional risk index; IFPI, inflammatory functional prognostic index; OACS, older adults with cancer sarcopenia; OS, overall survival.

**FIGURE 2:**
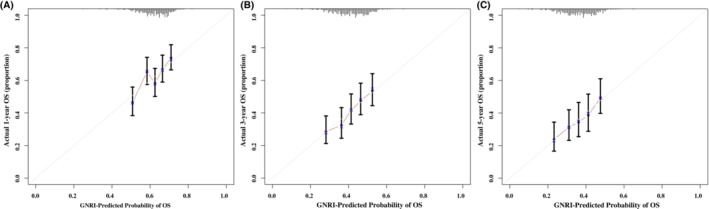
The 1‐, 3‐, and 5‐year calibration curves of the GNRI in OACS. GNRI, geriatric nutritional risk index; OACS, older adults with cancer sarcopenia; OS, overall survival.

When the GNRI score was used as a binary variable, 1‐, 3‐, and 5‐year multivariate survival analyses showed that, when compared with those patients with a high GNRI score (≥97.77), patients with a low GNRI score (<97.77) had a poorer prognosis [1 year: *p* = 0.032, HR (95% CI) = 1.773 (1.051–2.992); 3 years: *p* = 0.016, HR (95% CI) = 1.651 (1.100–2.479); 5 years: *p* = 0.018, HR (95% CI) = 1.602 (1.084–2.369)]. When the GNRI score was divided into four different risk groups, compared with those patients in the no risk group (>98), the 1‐, 3‐, and 5‐year prognosis of patients in the moderate (82 ~ 92) and severe risk (<82) groups were worse [1 year: *p* = 0.030, HR (95% CI) = 1.925 (1.062–3.491); 3 years: *p* = 0.006, HR (95% CI) = 1.903 (1.188–3.048); 5 years: *p* = 0.010, HR (95% CI) = 1.797 (1.141–2.830) and 1 year: *p* = 0.011, HR (95% CI) = 2.368 (1.118–5.015); 3 years: *p* = 0.004, HR (95% CI) = 2.250 (1.230–4.118); 5 years: *p* = 0.009, HR (95% CI) = 2.000 (1.115–3.590), respectively], while the values in the low risk group (92 ~ 98) were not statistically significant (all *p* > 0.05). As the GNRI score decreased, the patient's HR increased (1‐, 3‐, and 5‐year *p* values for trends were 0.018, 0.004, and 0.011, respectively) (Figure 1A,B, Figure S3, and Table 2). The sensitivity analysis showed the consistent results (Table S3). Interestingly, the 1‐, 3‐, and 5‐year calibration curves also indicated that the GNRI showed good consistency for prognosis predictions of OACS (Figure 2).

**TABLE 2:** Univariate and multivariate analysis of the OS in older adu with cancer sarcopenia

Variables	OS^#^		OS* (1 year)		OS* (3 years)		OS* (≥5 years)	
	Crude HR (95%CI)	Crude *p*	Adjusted HR (95%CI)	Adjusted *p*	Adjusted HR (95%CI)	Adjusted *p*	Adjusted HR (95%CI)	Adjusted *p*
By cut‐off								
GNRI ≥ 97.77	1		1		1		1	
GNRI < 97.77	2.230 1.542–3.226)	<0.001	1.816 (1.076–3.063)	0.025	1.678 (1.118–2.518)	0.013	1.627 (1.101–2.407)	0.015
By GNRI score								
No risk (98~)	1		1		1		1	
Low risk (92 ~ 98)	1.729 (1.114–2.683)	0.001	1.574 (0.898–2.759)	0.113	1.434 (0.919–2.238)	0.113	1.398 (0.912–2.143)	0.124
Moderate risk (82 ~ 92)	2.149 (1.432–3.227)	<0.001	1.933 (1.065–3.508)	0.030	1.931 (1.203–3.100)	0.006	1.817 (1.151–2.867)	0.010
Severe risk (~82)	2.994 (1.976–4.534)	<0.001	2.621 (1.249–5.503)	0.011	2.430 (1.333–4.430)	0.004	2.175 (1.219–3.883)	0.009
*p* for trend		<0.001		0.003		0.012		0.007

*Note*: OS^#^: Unadjusted; OS*: Adjusted for sex, radical resection, TNM stage, BMI, KPS, postoperative chemoradiotherapy, neutrophils, WBC, AST, ALT, serum albumin, comorbid disease(s), family history of cancer, hemoglobin, 30‐day mortality, reduced intake, reduced physical function, and PNI.

Abbreviations: ALT, alanine transaminase; AST, aspartate aminotransferase; BMI, body mass index; CI, confidence interval; GNRI, geriatric nutritional risk index; HR, hazards ratio; KPS, Karnofsky performance status; OS, overall survival; PNI, prognostic nutritional index; WBC, white blood cells.

**FIGURE 3:**
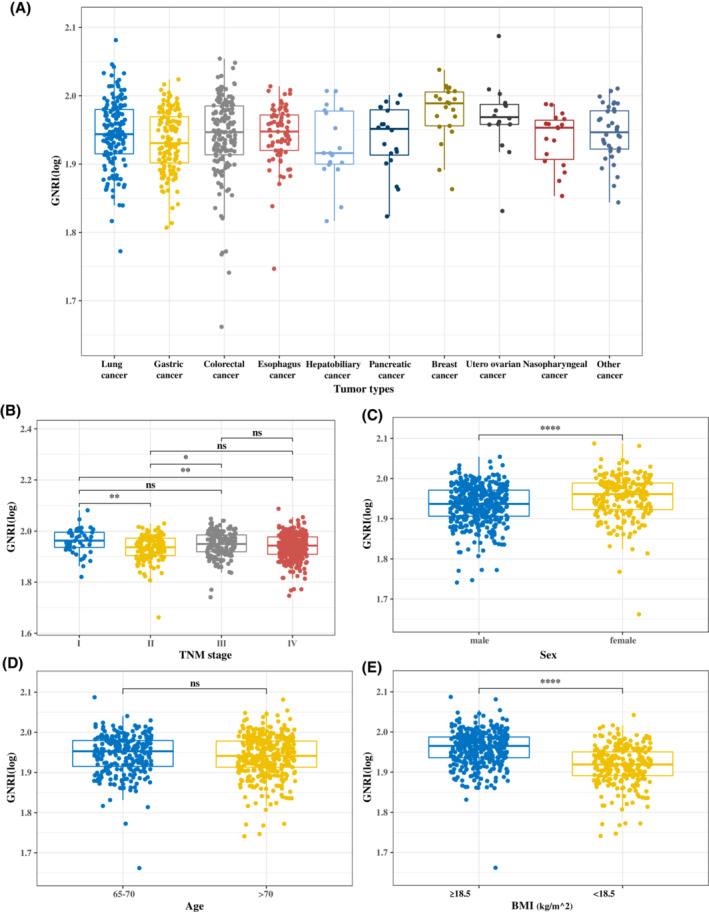
GNRI distribution in different groups. (A) tumor types; (B) TNM stage; (C) sex; (D) age; (E) BMI. BMI, body mass index; GNRI, geriatric nutritional risk index; TNM, tumor‐node‐metastasis.

The GNRI distribution curve results showed that lung cancer and digestive system tumors had lower GNRI scores than other tumors. With high TNM staging, the GNRI score also exhibited a downward trend. Additionally, the GNRI score of males and patients with a low BMI (<18.5) was higher than that of females and patients with a high BMI (≥18.5). No significant trends were observed in different age subgroups (Figure 3).

### 3.3 Stratified analysis

**FIGURE 4:**
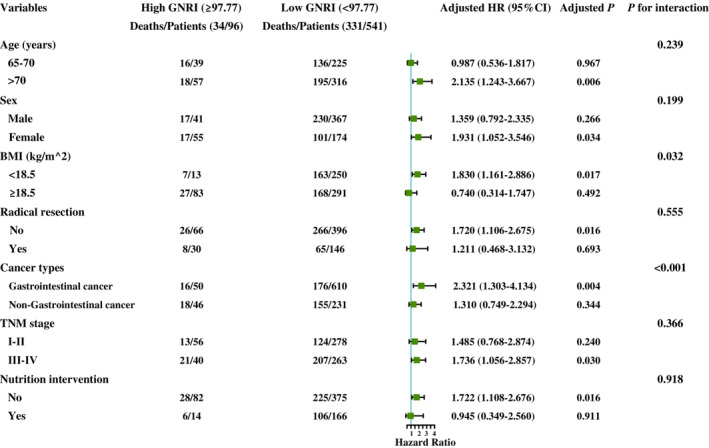
The stratification analysis of the GNRI in OACS. Adjusted for the sex, radical resection, TNM stage, BMI, KPS, postoperative chemoradiotherapy, neutrophil count, WBC count, AST, ALT, serum albumin, comorbid disease(s), family history of cancer, hemoglobin, 30‐day mortality, reduced food intake, reduced physical activity and PNI. ALT, alanine transaminase; AST, aspartate aminotransferase; BMI, body mass index; GNRI, geriatric nutritional risk index; KPS, Karnofsky performance status; OACS, older adults with cancer sarcopenia; PNI, prognostic nutritional index; WBC, white blood cells.

The stratified analysis showed that the presence of gastrointestinal cancer (*p* for interaction <0.001) and a low BMI (*p* for interaction = 0.032) were significantly associated with a low GNRI score. Additionally, we observed that a low GNRI score in older patients (>70 years), females, patients without radical resection or nutrition intervention, and patients with a TNM stage III–IV had a significantly higher mortality risk compared with patients with a high GNRI score (All *p* < 0.05) (Figure 4).

### 3.4 Construction and analysis of the IFPI

**FIGURE 5:**
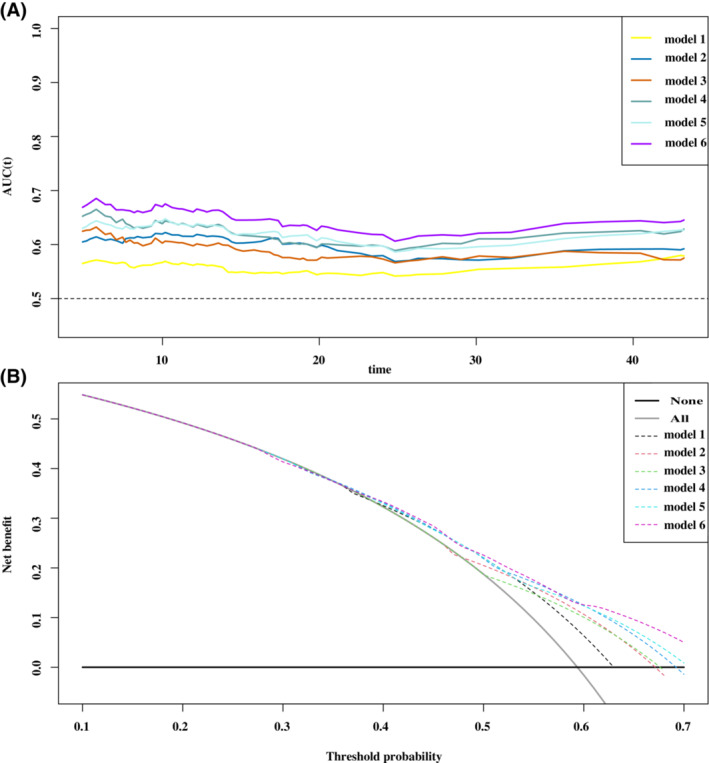
The DCA and prognostic ROC curves of different prognostic models. (A) the DCA; (B) ROC curves: Model 1, GNRI; Model 2, reduced food intake; Model 3, reduced physical activity; Model 4, GNRI + reduced food intake; Model 5, GNRI + reduced physical activity; and Model 6, GNRI + reduced food intake + reduced physical activity. DCA, decision curve analysis; GNRI, geriatric nutritional risk index; ROC, receiver operating characteristic.

The intake and physical activity and the inflammatory state were critical to the patient's survival (Figure S4). We compared the prognosis prediction capabilities of different model combinations: Model 1, GNRI; Model 2, reduced food intake; Model 3, reduced physical activity; Model 4, GNRI+ reduced food intake; Model 5, GNRI+ reduced physical activity; and Model 6, GNRI+ reduced food intake+ reduced physical activity. The prognostic receiver operator characteristic (ROC) curves and decision curve analysis (DCA) consistently showed that the prognostic capability of model 6 was better than that of the other models (Figure 5).

According to the survival curve of the IFPI in OACS, the IFPI score could be divided into three different risk groups; namely, the low‐risk, moderate‐risk, and high‐risk groups (Figure 1C,D). A similar result was also observed in the multivariate survival analysis of the IFPI in OACS based on the HR. Compared with patients with an IFPI score of 0, patients with a score of 4 [*p* = 0.004, HR (95% CI) = 2.675 (1.370–5.220)] and 5 [*p* = 0.016, HR (95% CI) = 2.474 (1.180–5.187)] had an increased risk of mortality (*p* for trends<0.001). Compared with patients in the low‐risk group, the moderate‐risk [*p* = 0.002, HR (95% CI) = 1.776 (1.232–2.559)] and high‐risk group patients [*p* < 0.001, HR (95% CI) = 2.616 (1.661–4.120)] had a significantly poorer prognosis (*p* for trends<0.001) (Table 3).

**TABLE 3:** The survival analysis of IFPI score

Variables	OS		OS^a^	
	Crude HR (95%CI)	Crude *p*	Adjusted HR (95%CI)	Adjusted *p*
IFPI score				
0	1		1	
1	1.154 (0.588–2.261)	0.677	0.990 (0.496–1.976)	0.978
2	1.951 (1.058–3.597)	0.032	1.682 (0.873–3.241)	0.120
3	2.035 (1.106–3.745)	0.022	1.896 (0.903–3.980)	0.091
4	3.311 (1.822–6.015)	<0.001	2.727 (1.145–6.494)	0.023
5	4.210 (2.281–7.773)	<0.001	2.872 (1.051–7.851)	0.040
*p* for trends		<0.001		0.011
IFPI score risk groups				
Low‐risk (0–1)	1		1	
Moderate‐risk^2^, ^3^	1.806 (1.277–2.554)	0.001	1.722 (1.154–2.57)	0.008
High‐risk^4^, ^5^	3.275 (2.33–4.604)	<0.001	2.509 (1.38–4.565)	0.003
*p* for trends		<0.001		0.003

Abbreviations: ALT, alanine transaminase; AST, aspartate aminotransferase; BMI, body mass index; CI, confidence interval; GNRI, geriatric nutritional risk index; HR, hazards ratio; IFPI: inflammatory functional prognostic index; KPS, Karnofsky performance status; OS, overall survival; PNI, prognostic nutritional index; WBC, white blood cells.

^a^ Adjusted for sex, radical resection, TNM stage, BMI, KPS, postoperative chemoradiotherapy, neutrophils, WBC, AST, ALT, serum albumin, comorbid disease(s), family history of cancer, hemoglobin, 30‐day mortality, reduced intake, reduced physical function, and PNI.

## 4 DISCUSSION

Sarcopenia can cause metabolic and endocrine abnormalities and contractile disorders and also affect the systemic metabolism and the immune or inflammatory response. Cancer can promote the release of pro‐inflammatory cytokines from tumor cells or the immune vascular system, leading to cancer cachexia and malnutrition in extreme cases.^19^ The elderly is a complex, vulnerable group worthy of attention and at high risk of nutritional problems, cachexia and sarcopenia. Due to aging, assessing the weight loss of older adults appears to be an obstacle to assessing their nutritional status. Our study analyzed the inflammation‐related indicator, GNRI, in OACS. Patients with a low GNRI score had a poorer prognosis than those with a high GNRI score. Some studies indicated the prognostic role of the GNRI in older adults with various cancers, including lung,^20^ prostate,^21^ head and neck,^22^ and gastrointestinal cancers.^23^, ^24^, ^25^ The GNRI score is a prognostic marker of inflammation consisting of albumin levels and body weight. The level of serum albumin is affected by the systemic inflammatory response (SIR),^12^ and hypoalbuminemia plays an important role in the development of cancer.^26^ The SIR is deeply involved in various carcinogenic and tumorigenic processes through host‐tumor interactions. Recently, Evans et al. suggested that hypoalbuminemia should be considered a chronic disease which was characterized by inflammation and closely related to adverse outcomes.^27^ When inflammation is present, the serum albumin concentration is reduced.^27^ Aging is often accompanied by the risk of muscle loss. Furthermore, elderly patients with cancer are in a state of high inflammation. The inflammatory state leads to an increase in cytokines produced by an increase in muscle catabolism. The imbalance of protein and energy metabolism in patients with cancer leaves the level of albumin in an imbalanced state. The ratio of PBW/IBW can reflect the patient's current nutritional status. If the patient is in a poor nutritional state, multiple body functions, such as immunity, digestive tract function, and wound healing, will be impaired. The lack of these functions will increase infection risk and postoperative complications risk, and the immunosuppressed state will lead to insufficient anti‐tumor immune response. The PBW/IBW ratio can reflect the frailty, cachexia, and a worse prognosis of older adults with cancer. In general, the level of albumin and weight status provide the GNRI a greater prognostic value than a single indicator. Similarly, our analysis of the GNRI also found that its 1‐, 3‐, and 5‐year prognostic performance in OACS showed good consistency.

In subgroup analyses, we found that a low BMI (*p* for interaction = 0.032) and the presence of gastrointestinal cancer (*p* for interaction <0.001) have a strong correlation with a low GNRI score. The BMI can reflect the patient's nutritional status and the result of negative energy balance. Among elderly community residents, a BMI less than 22 was associated with a higher 1‐year mortality rate and the poor functional status.^28^ Notably, previous studies showed that BMI was related to skeletal muscle reduction.^29^, ^30^ In our study, patients with advanced tumors often had dietary decline. We hypothesized that the patient's cachexia‐anorexia syndrome further aggravated the patient's negative energy and inflammatory states, worsened their nutritional status, and further aggravated muscle loss. Many patients with gastrointestinal cancer will have obstruction, such as colon cancer intestinal obstruction, and therefore experience secondary dietary decline and nutrient absorption disorders.

In our study, people with reduced food intake and reduced physical activity had a large proportion of high GNRI results. Therefore, we analyzed the combined effect of these three factors and divided the population into three categories based on the results of survival analysis: Low risk (those without reduced intake and physical activity and with a high GNRI score), medium risk (those who did not fall into the high‐ or low‐risk categories), and high risk (those with reduced intake and physical activity and a low GNRI score). Compared with low‐risk patients, high‐risk patients had a higher mortality rate. In aging and chronic diseases, muscle loss and fat changes have different metabolic trajectories.^8^ Appetite regulation and physical activity affect energy balance and changes in body fat mass.^8^ Inflammation can induce anorexia and fat loss, accompanied by muscle loss. In other cases, even though systemic inflammation is activated, appetite is maintained, resulting in skeletal muscle reduction with a normal or increased BMI. The physical activity function of OACS cannot be ignored. Inactivity can lead to muscle loss and increase the fat tissue in aging and disease.^8^ Exercise can improve muscle mass and reduce inflammation, which can inhibit muscle catabolism by increasing protein synthesis and reducing protein degradation, and repeated exercise can weaken the cellular response to inflammatory stimuli and pro‐inflammatory cytokines.^31^ IL‐6 has anti‐inflammatory effects, inhibits pro‐inflammatory cytokines (tumor necrosis factor [TNF]‐α and IL‐1) and activates the production of anti‐inflammatory factors (IL‐1ra and IL‐10).^32^ The anti‐inflammatory role of exercise may have the ability to reduce systemic inflammation and inflammatory cytokines, thereby weakening their role in mediating the wasting process in cachexia. Conversely, insufficient muscle strength may contribute to a local muscle inflammation, which further leads to damage and drives systemic inflammation. Aging, systemic inflammation, physical activity, feeding function, muscle mass, and strength form a cascade reaction cycle. This cyclic mechanism, in turn, can enhance the aggressiveness of the tumor or reduce the response to treatment, impairing the transition to survival. Age‐related muscle atrophy and persistent muscle atrophy caused by cancer cachexia may be a metabolic challenge that older adults cannot cope with using skeletal muscles. The treatment of OACS should be comprehensively evaluated and intervention measures for many aspects are necessary.

Our study has some limitations that need to be strengthened. First, reduced food intake and decreased physical activity data were obtained through questionnaire surveys to evaluate the subjective feelings or manifestations in patients. Specific evaluation or measurement of food intake and physical activity ability is necessary. Second, clinical indicators, such as C‐reactive protein and IL‐6, need to be included. Finally, a large sample, increased number of centers or hospitals, and regional populations should be included for further confirmation.

## 5 CONCLUSION

The GNRI score is a very simple and objective inflammatory prognostic indicator for OACS. It can be used as a short‐term and long‐term prognostic indicator. Evaluating the combination of the inflammatory GNRI, diet status, and physical activity status can improve patient survival prediction.

## AUTHOR CONTRIBUTIONS

Guo‐Tian Ruan wrote the manuscript. Guo‐Tian Ruan, Hai‐Lun Xie, and He‐Yang Zhang analyzed and interpreted the patient data, Guo‐Tian Ruan, Hai‐Lun Xie, He‐Yang Zhang, and Han‐Ping Shi made substantial contributions to the conception, design, and intellectual content of the studies. All authors read and approved the final manuscript.

## FUNDING INFORMATION

This work was supported by the National Key Research and Development Program [grant number 2017YFC1309200] and the Beijing Municipal Science and Technology Commission [grant number SCW2018‐06].

## CONFLICT OF INTEREST

Non‐financial competing interests.

## ETHICS APPROVAL

This study followed the Helsinki declaration. All participants signed an informed consent form and this study was approved by the Institutional Review Board of each hospital (Registration number: ChiCTR1800020329).

## PATIENT CONSENT

Not applicable.

## Supporting information


Data S1

Figure S1

Figure S2

Figure S3

Figure S4

Table S1

Table S2

Table S3

## Data Availability

The datasets used and/or analyzed during the current study are available from the corresponding author on reasonable request.
