# Supplementary material for: Inflammatory geriatric nutritional risk index stratified the survival of older adults with cancer sarcopenia
Source: Cancer Med. 2022 Nov 29;12(6):6558–70. doi: 10.1002/cam4.5427 (PMC10067041; doi:10.1002/cam4.5427)
Supplement: Supplementary file 1 — Data S1 [file CAM4-12-6558-s004.docx]

**Supplementary figure legends**

**Figure S1** Flowchart of patient selection for this study.

**Figure S2** The optimal cut-off value of the geriatric nutritional risk index according to the results of standardized log-rank statistics.

**Figure S3** Cumulative mortality risk curve of GNRI.

**Figure S4** The Kaplan–Meier survival curves of reduced food intake and reduced physical activity of overall survival in elderly patients with cancer sarcopenia.

**Table S1**. The names of all participating hospitals.

**Table S2** Univariate and multivariate analyses of OS in elderly patients with cancer sarcopenia

**Table S3** Sensitivity analysis of the OS in elderly patients with cancer sarcopenia
