# Supplementary figures and images for: Inflammatory geriatric nutritional risk index stratified the survival of older adults with cancer sarcopenia
Source: Cancer Med. 2022 Nov 29;12(6):6558–70. doi: 10.1002/cam4.5427 (PMC10067041; doi:10.1002/cam4.5427)

# Figure S1

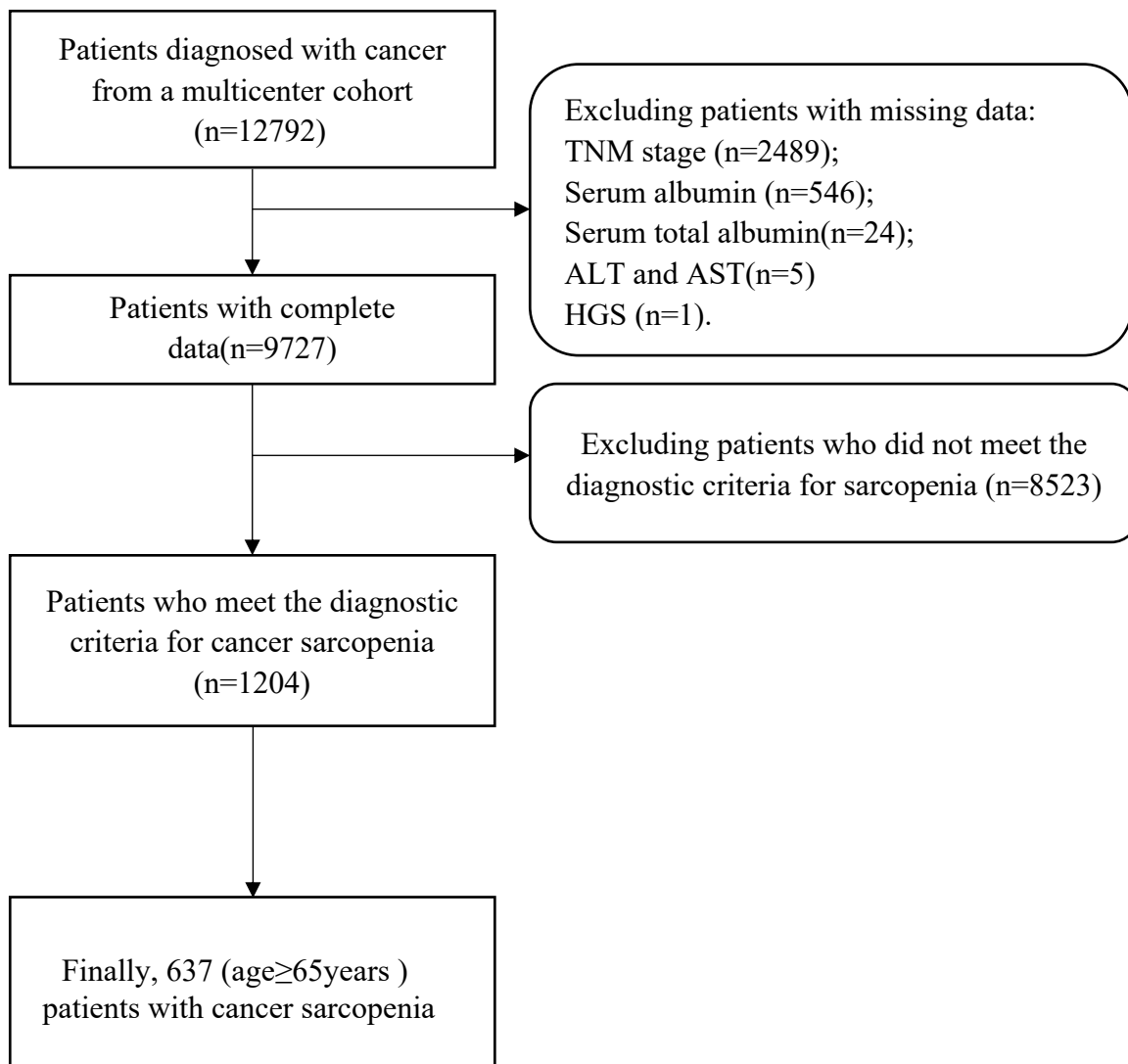

Supplement: Supplementary file 2 — Figure S1 [file CAM4-12-6558-s008.pdf]

Figure S2

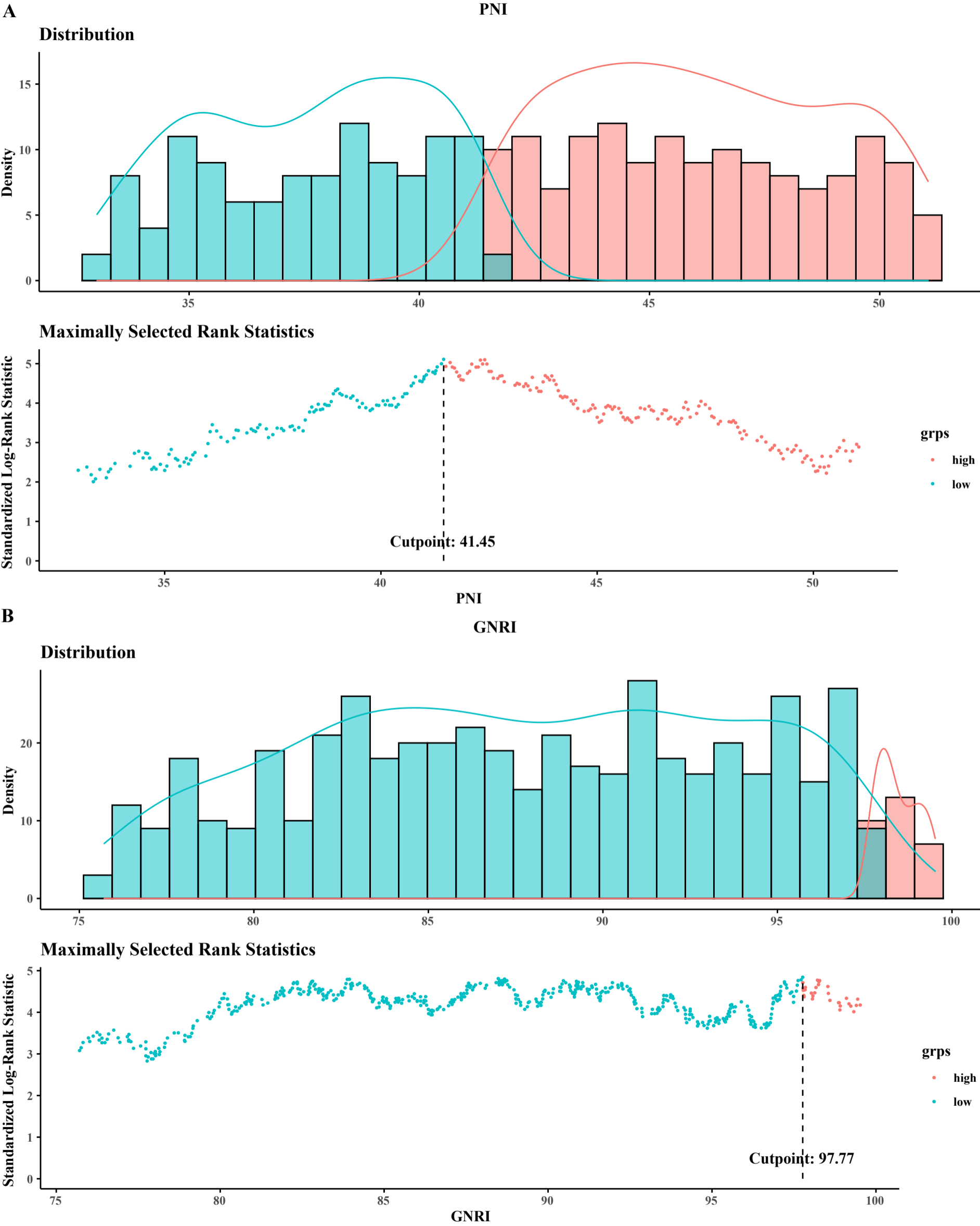

Supplement: Supplementary file 3 — Figure S2 [file CAM4-12-6558-s003.pdf]

Figure S3

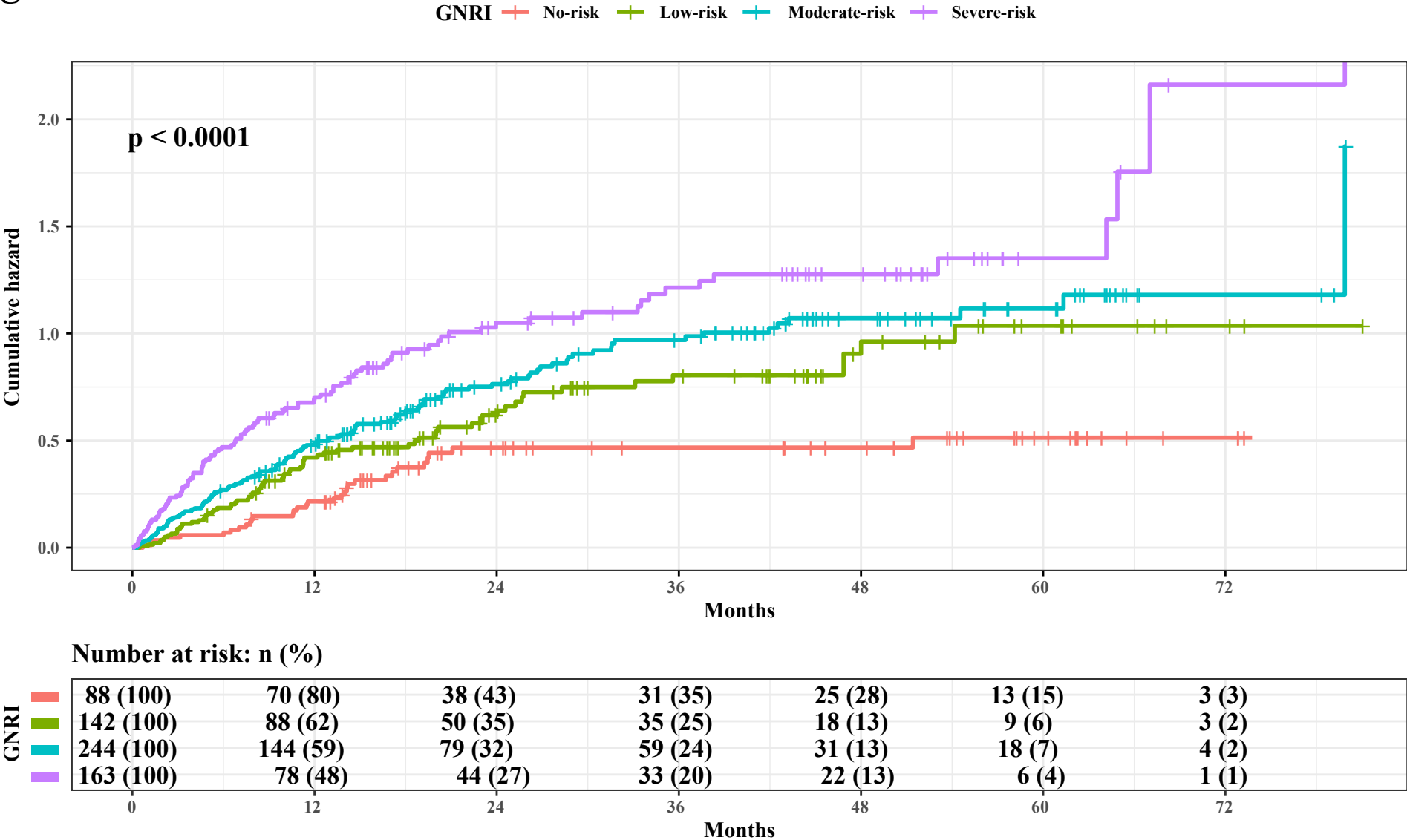

Supplement: Supplementary file 4 — Figure S3 [file CAM4-12-6558-s006.pdf]

Figure S4

A

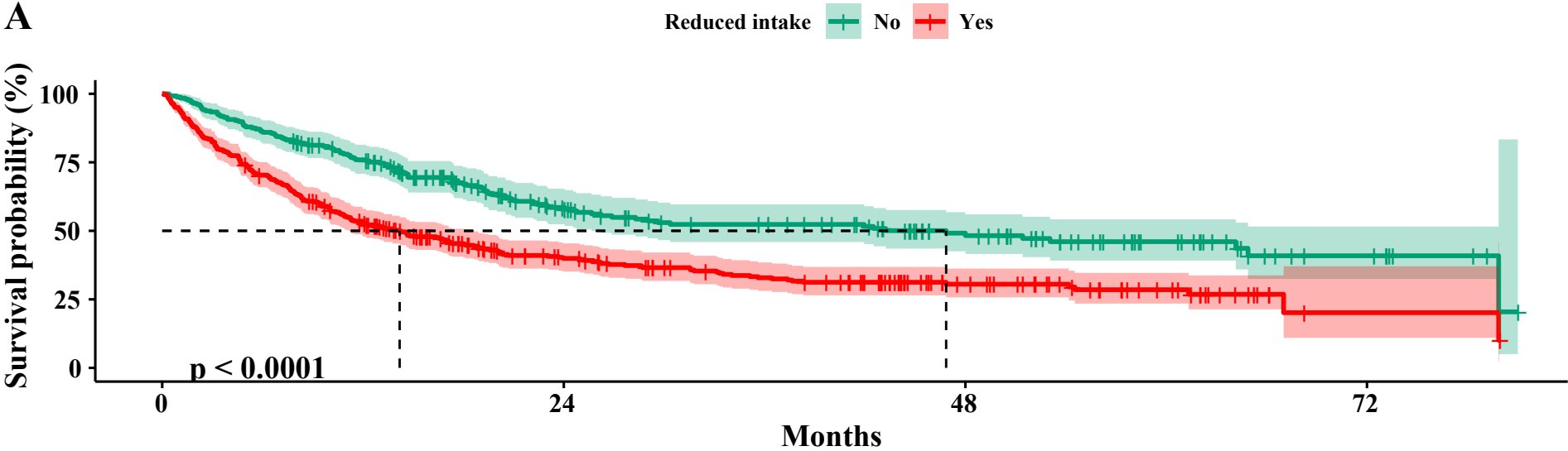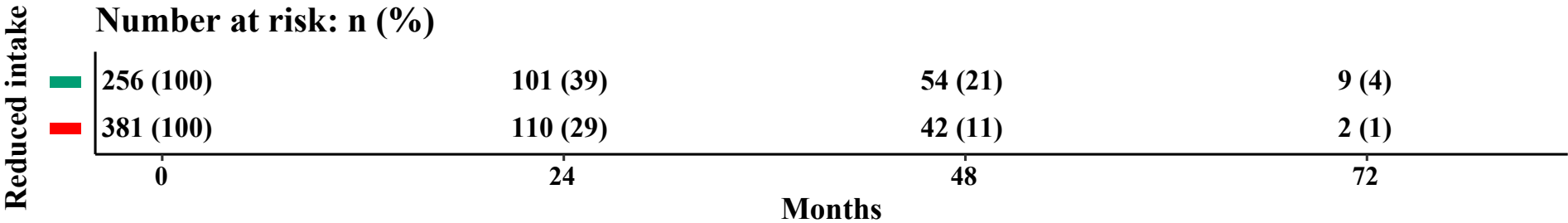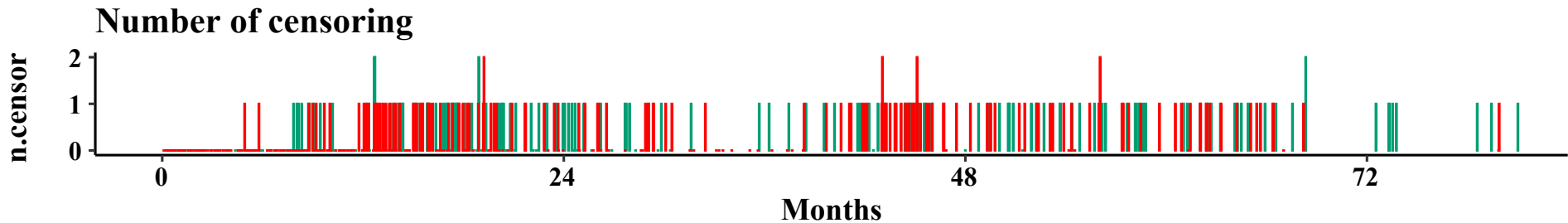

B

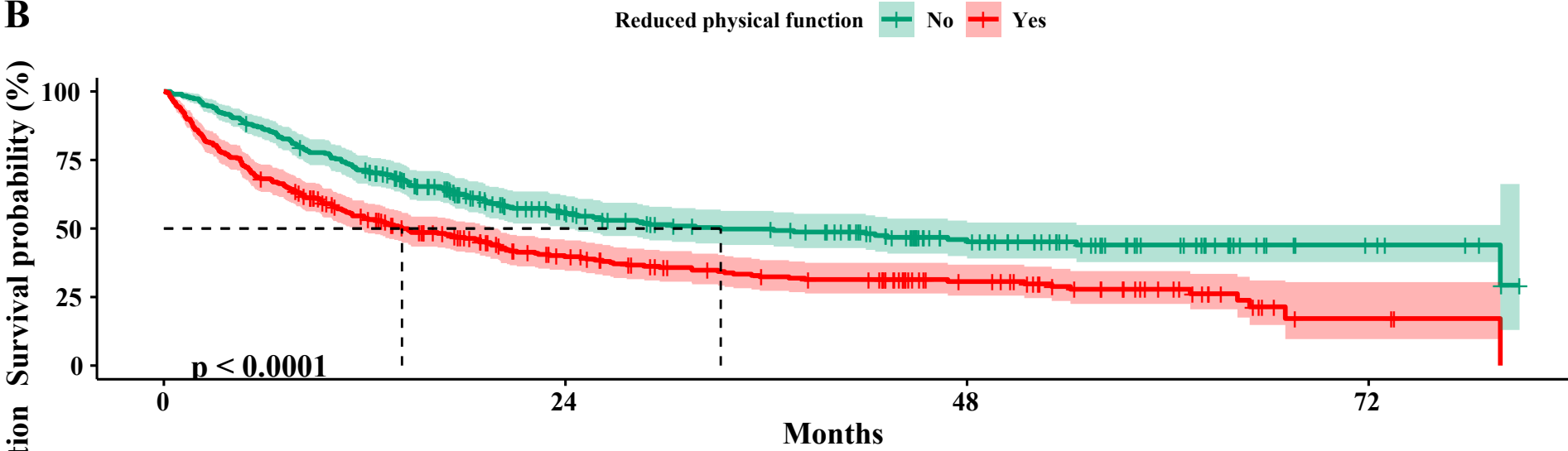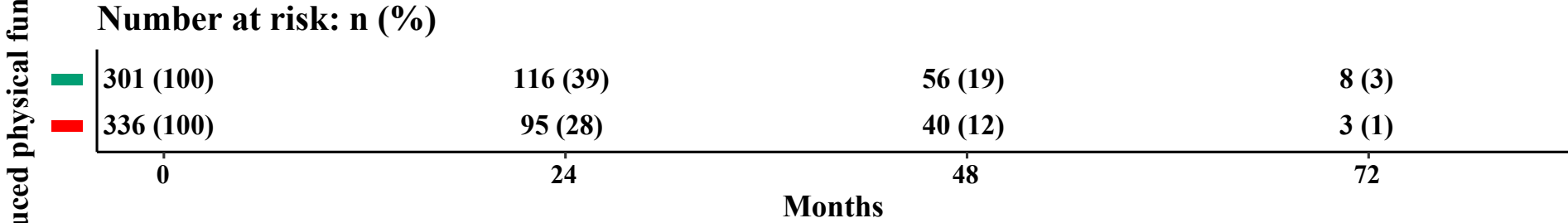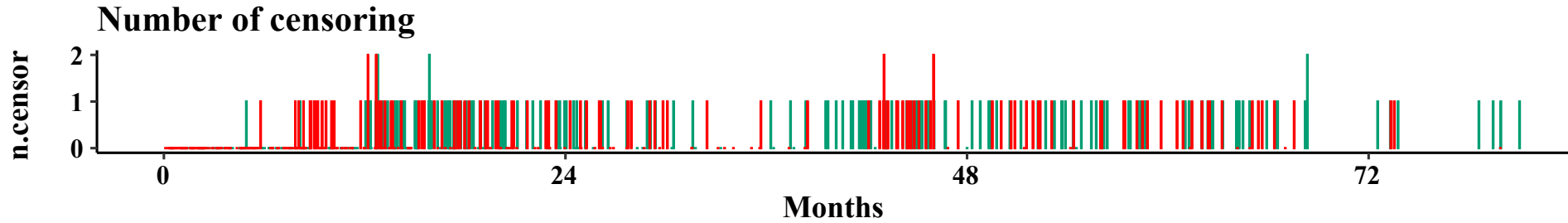

Supplement: Supplementary file 5 — Figure S4 [file CAM4-12-6558-s007.pdf]
