# Supplementary material for: Inflammatory geriatric nutritional risk index stratified the survival of older adults with cancer sarcopenia
Source: Cancer Med. 2022 Nov 29;12(6):6558–70. doi: 10.1002/cam4.5427 (PMC10067041; doi:10.1002/cam4.5427)
Supplement: Supplementary file 6 — Table S1 [file CAM4-12-6558-s002.docx]

**Table S1.** The names of all participating hospitals.

| Number | Hospitals |
| --- | --- |
| 1 | Anhui Cancer Hospital |
| 2 | Beijing Cancer Hospital |
| 3 | Affiliated Hospital of Chengde Medical College |
| 4 | Tangdu Hospital of Fourth Military Medical University |
| 5 | Foshan First People Hospital |
| 6 | Fujian Cancer Hospital |
| 7 | Guangdong Provincial People Hospital |
| 8 | Guangxi Guigang People Hospital |
| 9 | Guangxi Medical University Affiliated Cancer Hospital |
| 10 | The First Affiliated Hospital of Guangxi Medical University |
| 11 | Guangxi Zhuang People Hospital |
| 12 | Affiliated Cancer Hospital of Zunyi Medical College, Guizhou Province |
| 13 | Affiliated Cancer Hospital of Harbin Medical University |
| 14 | The Fourth Affiliated Hospital of Harbin Medical University |
| 15 | Hebei Provincial People's Hospital |
| 16 | The Second Affiliated Hospital of Hebei Medical University |
| 17 | The Fourth Affiliated Hospital of Hebei Medical University |
| 18 | The First Affiliated Hospital of Hebei Medical University |
| 19 | Huizhou Central People Hospital |
| 20 | Bethune First Hospital of Jilin University |
| 21 | The Third Affiliated Hospital of Kunming Medical University |
| 22 | The First Affiliated Hospital of Kunming Medical University |
| 23 | Liaoning Cancer Hospital |
| 24 | The First Hospital of Shanxi Medical University |
| 25 | Shanghai Ruijin Hospital |
| 26 | Shanghai Tenth People Hospital |
| 27 | Beijing Shijitan Hospital Affiliated to Capital Medical University |
| 28 | West China Hospital of Sichuan University |
| 29 | Sichuan Cancer Hospital |
| 30 | Tianjin Medical University Cancer Hospital |
| 31 | Wuhan Tongji Hospital |
| 32 | Xijing Hospital |
| 33 | The First People Hospital of Kashgar, Xinjiang |
| 34 | Xingtai People's Hospital |
| 35 | Yunnan Cancer Hospital |
| 36 | Yuncheng Central Hospital |
| 37 | The First Affiliated Hospital of Zhejiang University |
| 38 | The Second Affiliated Medical Hospital of Zhejiang University |
| 39 | Zhejiang First Hospital |
| 40 | Zhejiang People's Hospital |
| 41 | Department of Integrated Traditional Chinese and Western Medicine, Zhejiang Cancer Hospital |
| 42 | Cancer Hospital of Chinese Academy of Medical Sciences |
