# Supplementary material for: Inflammatory geriatric nutritional risk index stratified the survival of older adults with cancer sarcopenia
Source: Cancer Med. 2022 Nov 29;12(6):6558–70. doi: 10.1002/cam4.5427 (PMC10067041; doi:10.1002/cam4.5427)
Supplement: Supplementary file 7 — Table S2 [file CAM4-12-6558-s001.docx]

**Table S2 Univariate analyses of OS in older adults with cancer sarcopenia.**

|  | OS | |  |
| --- | --- | --- | --- |
| Variables | Crude HR (95%CI) | Crude *P* |  |
| Age (year) |  |  |  |
| 65-70 | 1 |  |  |
| >70 | 0.956 (0.776-1.177) | 0.671 |  |
| Sex |  |  |  |
| Male | 1 |  |  |
| Female | 0.753 (0.604-0.937) | 0.011 |  |
| Tea consumption |  |  |  |
| No | 1 |  |  |
| Yes | 1.016 (0.802-1.287) | 0.894 |  |
| Alcohol consumption |  |  |  |
| No | 1 |  |  |
| Yes | 1.041 (0.800-1.355) | 0.766 |  |
| Smoking |  |  |  |
| No | 1 |  |  |
| Yes | 1.068 (0.869-1.311) | 0.533 |  |
| Family history of cancer |  |  |  |
| No | 1 |  |  |
| Yes | 1.337 (1.003-1.782) | 0.047 |  |
| Comorbid disease(s) |  |  |  |
| 0 | 1 |  |  |
| 1 | 1.319 (1.041-1.671) | 0.022 |  |
| 2 | 1.416 (1.017-1.971) | 0.040 |  |
| 3 or more | 1.328 (0.861-2.047) | 0.199 |  |
| BMI (kg/m^2) |  |  |  |
| <18.5 | 1 |  |  |
| ≥18.5 | 0.686 (0.559-0.843) | <0.001 |  |
| Neoadjuvant chemoradiotherapy |  |  |  |
| No | 1 |  |  |
| Yes | 0.733 (0.364-1.477) | 0.385 |  |
| Postoperative chemoradiotherapy | |  |  |
| No | 1 |  |  |
| Yes | 1.706 (1.388-2.097) | <0.001 |  |
| Radical resection |  |  |  |
| No | 1 |  |  |
| Yes | 0.433 (0.334-0.561) | <0.001 |  |
| TNM stage |  |  |  |
| Ⅰ | 1 |  |  |
| Ⅱ | 1.497 (0.773-2.899) | 0.231 |  |
| Ⅲ | 2.562 (1.365-4.809) | 0.003 |  |
| Ⅳ | 6.105 (3.325-11.207) | <0.001 |  |
| KPS |  |  |  |
| ≥80 | 1 |  |  |
| 50-80 | 1.737 (1.361-2.217) | <0.001 |  |
| <50 | 2.417 (1.750-3.340) | <0.001 |  |
| Serum total protein (g/L) |  |  |  |
| <60 | 1 |  |  |
| ≥60 | 0.940 (0.743-1.191) | 0.610 |  |
| Serum albumin (g/L) |  |  |  |
| <35 | 1 |  |  |
| ≥35 | 0.623 (0.507-0.765) | <0.001 |  |
| AST (U/L) |  |  |  |
| ≤40 | 1 |  |  |
| >40 | 1.403 (1.061-1.854) | 0.017 |  |
| ALT (U/L) |  |  |  |
| ≤50 | 1 |  |  |
| >50 | 1.484 (1.068-2.062) | 0.019 |  |
| Hemoglobin (g/L) |  |  |  |
| Male<110 and Female<120 | 1 |  |  |
| Male≥110 and Female≥120 | 1.313 (1.068-1.614) | 0.010 |  |
| WBC (×10^9/L) |  |  |  |
| ≤10 | 1 |  |  |
| >10 | 1.531 (1.158-2.025) | 0.003 |  |
| Neutrophils (×10^9/L) |  |  |  |
| ≤8 | 1 |  |  |
| >8 | 1.810 (1.376-2.381) | <0.001 |  |
| Lymphocytes (×10^9/L) |  |  |  |
| ≤1.5 | 1 |  |  |
| >1.5 | 0.845 (0.681-1.048) | 0.125 |  |
| Platelet (×10^9/L) |  |  |  |
| ≤160 | 1 |  |  |
| >160 | 1.169 (0.910-1.503) | 0.221 |  |
| Nutrition intervention |  |  |  |
| No | 1 |  |  |
| Yes | 1.097 (0.877-1.372) | 0.417 |  |
| Reduced physical function |  |  |  |
| No | 1 |  |  |
| Yes | 1.938 (1.488-2.524) | <0.001 |  |
| Reduced intake |  |  |  |
| No | 1 |  |  |
| Yes | 1.831 (1.469-2.283) | <0.001 |  |
| 30-day mortality |  |  |  |
| No | 1 |  |  |
| Yes | 7.196 (4.516-11.467) | <0.001 |  |
| PNI |  |  |  |
| ≥42.425 | 1 |  |  |
| <42.425 | 1.772 (1.455-2.158) | <0.001 |  |

Notes: OS, Overall Survival; HR, Hazards Ratio; CI, Confidence Interval; BMI: Body Mass Index; KPS, Karnofsky Performance Status; AST: Aspartate Aminotransferase; ALT: Alanine Transaminase; WBC: White Blood Cells; GNRI: Geriatric Nutritional Risk Index; PNI: Prognostic Nutritional Index.
