# Supplementary material for: Inflammatory geriatric nutritional risk index stratified the survival of older adults with cancer sarcopenia
Source: Cancer Med. 2022 Nov 29;12(6):6558–70. doi: 10.1002/cam4.5427 (PMC10067041; doi:10.1002/cam4.5427)
Supplement: Supplementary file 8 — Table S3 [file CAM4-12-6558-s005.docx]

**Table S3 Sensitivity analysis of the OS in older adults with cancer sarcopenia.**

| Variables | OS**^#^** | |  | OS***** (1 year) | |  | OS***** (3 years) | |  | OS***** (≥5 years) | |
| --- | --- | --- | --- | --- | --- | --- | --- | --- | --- | --- | --- |
|  | Crude HR (95%CI) | Crude P |  | Adjusted HR (95%CI) | Adjusted P |  | Adjusted HR (95%CI) | Adjusted P |  | Adjusted HR (95%CI) | Adjusted P |
| By cut-off |  |  |  |  |  |  |  |  |  |  |  |
| GNRI≥97.77 | 1 |  |  | 1 |  |  | 1 |  |  | 1 |  |
| GNRI <97.77 | 2.057 (1.4-3.022) | <0.001 |  | 2.164 (1.167-4.011) | 0.014 |  | 1.779 (1.137-2.782) | 0.012 |  | 1.699 (1.108-2.606) | 0.015 |
| By GNRI score |  |  |  |  |  |  |  |  |  |  |  |
| No risk (98~) | 1 |  |  | 1 |  |  | 1 |  |  | 1 |  |
| Low risk (92~98) | 1.680 (1.067-2.644) | 0.025 |  | 1.821 (0.955-3.472) | 0.069 |  | 1.508 (0.928-2.451) | 0.097 |  | 1.462 (0.92-2.324) | 0.108 |
| Moderate risk (82~92) | 1.931 (1.268-2.941) | 0.002 |  | 2.128 (1.055-4.29) | 0.035 |  | 1.966 (1.166-3.313) | 0.011 |  | 1.826 (1.107-3.013) | 0.018 |
| Severe risk (~82) | 2.509 (1.622-3.88) | <0.001 |  | 3.344 (1.372-8.149) | 0.008 |  | 2.674 (1.363-5.248) | 0.004 |  | 2.357(1.236-4.493) | 0.009 |
| *P* for trend |  | <0.001 |  |  | 0.009 |  |  | 0.004 |  |  | 0.009 |

Notes: The sensitivity analysis was to exclude patients who died within 3 months. OS**^#^**: Unadjusted; OS*****: Adjusted for sex, radical resection, TNM stage, BMI, KPS, postoperative chemoradiotherapy, neutrophils, WBC, AST, ALT, serum albumin, comorbid disease(s), family history of cancer, hemoglobin, 30-day mortality, reduced intake, reduced physical function, and PNI. OS, Overall Survival; HR, Hazards Ratio; CI, Confidence Interval; BMI: Body Mass Index; KPS, Karnofsky Performance Status; AST: aspartate Aminotransferase; ALT: alanine Transaminase; WBC: White Blood Cells; GNRI: Geriatric Nutritional Risk Index; PNI: Prognostic Nutritional Index.
